# The Regulation of Immune Responses by DC Derived Type I IFN

**DOI:** 10.3389/fimmu.2013.00094

**Published:** 2013-04-22

**Authors:** Dennis Ng, Jennifer L. Gommerman

**Affiliations:** ^1^Department of Immunology, University of TorontoToronto, ON, Canada

**Keywords:** lymphotoxin, tumor necrosis factor, toll-like receptors, dendritic cells

## Abstract

Our immune system bears the tremendous task of mounting effective anti-microbial responses whilst maintaining immunoregulatory functions to avoid autoimmunity. In order to quickly respond to pathogens, Dendritic cells (DC) are armed with pattern recognition receptors (PRRs), allowing them to recognize highly conserved pathogen-associated molecular patterns (PAMPs) that are uniquely expressed by invading microbes. PRR activation can trigger DCs to release the pleiotropic cytokine, Type I interferons (IFN), which facilitates various biological functions in different immune cell types. In this review, we will discuss the classical PRR-induced Type I IFN response in DCs as well as describe a novel mechanism for Type I IFN induction by the tumor-necrosis factor receptor superfamily (TNFRSF) members, TNFR-1 and lymphotoxin-β receptor (LTβR). While PRR activation during viral infection, produces large amounts of Type I IFN in a relative short period of time, TNFRSF-induced Type I IFN expression is modest with gradual kinetics. Type I IFN can exert pro-inflammatory effects, but in some cases it also facilitates immune-regulatory functions. Therefore, DCs are important regulators of immune responses by carefully modulating Type I IFN expression.

## Introduction

Dendritic cells (DCs) play a critical role in bridging the innate immune response with the adaptive immune system. DCs reside throughout the body, particularly enriched in secondary lymphoid organs (SLOs), intestinal lamina propria, and the epidermal layer of the skin, and they constantly sample antigen to maintain balance between immunity and tolerance. Infection triggers pattern recognition receptors (PRR) activation, promoting DC maturation, and the production of inflammatory cytokines including TNF-α, interleukin (IL)-1, IL-6, IL-12, and Type I interferons (IFN). Upon activation fully mature DCs migrate to SLOs where they interact with naive T cells, resulting in the activation of the adaptive immune response. DCs can be broadly categorized into conventional DCs (cDCs) and plasmacytoid DCs (pDCs). cDCs are comprised of different subsets governed by differential expression of CD4 and CD8α (Villadangos and Schnorrer, 2007; Hashimoto et al., 2011). cDCs are highly efficient at presenting external antigens through the major histocompatibility complex (MHC) class I molecule to CD8+ T cells for the generation of cytotoxic T lymphocytes (CTLs), a process known as cross-presentation that plays an important role in pathological contexts including autoimmune disease, allograft rejection, and tumor immunity (Carbone and Bevan, 1990; Den Haan et al., 2000; Pooley et al., 2001). pDCs are also capable of capturing, processing, and presenting antigens to T cells (Sapoznikov et al., 2007; Young et al., 2008), but in comparison to cDCs, they possess more restricted antigen uptake and lower levels of co-stimulatory molecules and MHC (Colonna et al., 2004; Dalgaard et al., 2005; Young et al., 2008). Instead, pDCs patrol between blood vessels and the lymphatic system, and when they encounter viruses, they are capable of secreting very large amounts of Type I IFN in a short period of time as a strategy for viral control (Siegal, 1999; Colonna et al., 2004). Therefore, together cDCs and pDCs provide both distinct and overlapping contributions towards host defense against pathogens.

IFNs were first discovered to be an important anti-viral factor that interferes with viral replication in mammalian cells. There are three classes of IFN (Type I, II, and III) categorized based on their structural homology and the specific receptor with which they associate (Platanias, 2005). The Type I IFN family is very diverse and includes a single IFN-β member, numerous IFN-α variants (13 in human and 14 in mouse), and the lesser known IFN-ε, -κ, -ω, and -δ (Sen, 2001). Despite this diversity, all Type I IFNs bind exclusively to the interferon-α receptor (IFNAR). Upon receptor activation, IFNAR1 and IFNAR2 dimerize and phosphorylate the Janus Kinase family members TYK2 and JAK1. Activated TYK2 and JAK1 phosphorylate STAT1 and STAT2, and together they bind IRF9 to form a trimeric transcription factor, ISGF3. ISGF3 translocates into the nucleus and interacts with the ISRE elements to activate IFN-related gene transcription (Platanias, 2005). Type I IFNs are secreted by various cell types including fibroblasts, epithelial cells, innate immune cells, and lymphocytes, and they represent a key initiating factor against viral infections. In this review, we will describe the conventional mechanism of Type I IFN production by cDCs, and for brevity will restrict ourselves to virus infection scenarios. In addition, we will discuss a novel mechanism of Type I IFN induction that is triggered by tumor-necrosis factor receptor superfamily (TNFRSF) members such as TNFR-1 and lymphotoxin-β receptor (LTβR), independent of PRR activation. Finally, we will contextualize the versatile role of Type I IFN in tuning T cell responses in different contexts such as responses to replicating pathogens as opposed to cell-associated protein antigens.

## PRR-Induced Type I IFN in Response to Viral Infection

Our immune system is in a constant evolutionary battle with pathogens that has played out through millennia. In order to combat viral infections, the immune system relies on detection tools, in particular PRR, that elicit potent Type I IFN expression.

### Toll-like receptors

Thus far, 13 mammalian TLRs have been identified (10 in human and 12 in mice) and each TLR recognizes a specific class of microbial pathogen-associated molecular patterns (PAMPs) that triggers distinct responses (Casanova et al., 2011). TLRs belong to the IL-1 receptor family, and all of them contain a common cytoplasmic domain referred to as the toll-interleukin-1 receptor (TIR) domain. Upon activation, the TIR domain recruits TIR-associated adapter molecules including MyD88, TRIF, TRAM, and TIRAP that mediate various downstream signaling pathways. TLRs are mainly expressed in immune cells such as neutrophils, macrophages, DC, and some lymphocytes, while non-immune cells such as fibroblasts and intestinal epithelial cells express a more restricted sets of TLRs (Reynolds et al., 2010; Akira, 2011). The majority of TLRs are found on the cell membrane with the exception of TLR3, 7–9, and 11–13, which are expressed within intracellular endosomes. These TLRs are associated with the detection of viral, bacterial, and parasitic nucleic acids (Meylan and Tschopp, 2006; Stetson and Medzhitov, 2006), and their sub-cellular compartmentalization allows the immune system to distinguish self from non-self antigens (Barton et al., 2006). Under certain circumstances however, host nucleic acids can be mistaken as non-self resulting in autoimmune diseases. For example, in the case of systemic lupus erythematosis, complexes of autoreactive antibodies and host nucleic acids can trigger the activation of endosomal TLRs in pDCs, resulting in uncontrolled production of Type I IFN and disease pathology (Elkon and Stone, 2011).

Conventional DCs and pDCs express different sets of TLRs that facilitate their specialized function. Human pDCs lack TLR3 but express high levels of TLR7, TLR8, and TLR9, and they are responsible for the production of large amounts of Type I IFN in response to viral infections (Hornung et al., 2002). The signaling pathway downstream of TLR7, 8, and 9 is mediated through the key adaptor molecule MyD88 (Takeda and Akira, 2005). Upon activation, MyD88 recruits IRAK4 and IRAK1 to the receptors, and through interaction of their death domains, IRAK1 becomes phosphorylated. Activated IRAK1 further recruits and activates the constitutively expressed IRF7 in pDC, resulting in the production of Type I IFN (Coccia et al., 2004; Asselin-Paturel and Trinchieri, 2005; Honda et al., 2005).

**Figure 1:**
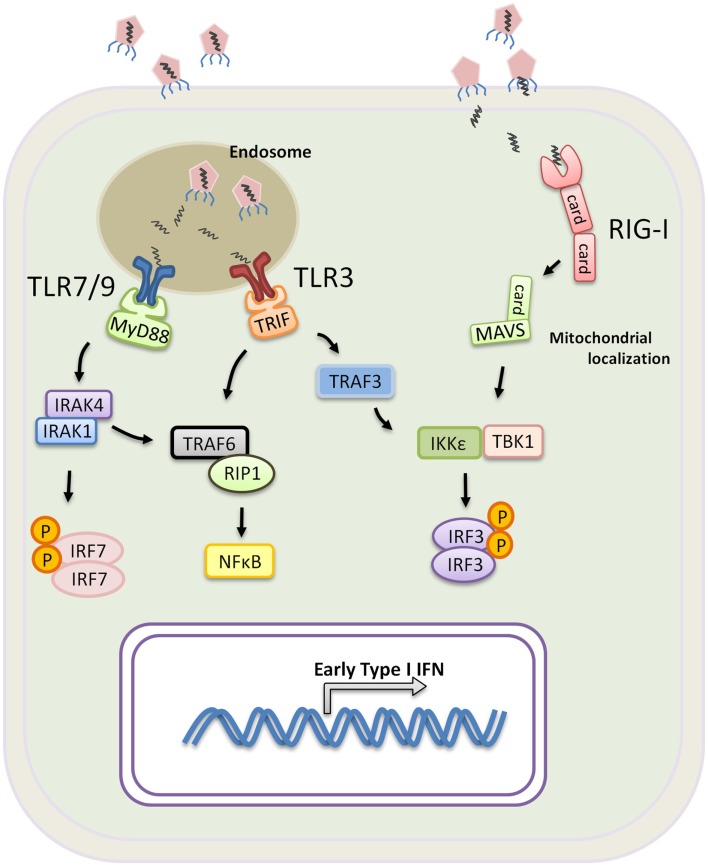
**Induction of Type I IFN by PRR during a viral infection**. TLR3, 7, and 9 are mainly expressed within the endosomes of innate immune cells. Virus or virus-infected cells are taken up by macrophages or DCs, and the viral nucleic molecules are exposed upon endosomal acidification. Activation of TLR7 and 9 requires signaling through MyD88 and recruitment of IRAK4, IRAK1, and IRF7. IRF7 becomes phosphorylated and translocates into the nucleus upon dimerization resulting in transcription of Type I IFN genes. TLR3 signals exclusively through Trif which binds TRAF6 and recruits RIP1 for NF-κB activation. Trif also binds TRAF3 leading to TRAF3 K63-linked ubiquintination, facilitating the recruitment of TBK1, IKKε and IRF3 for IRF3 phosphorylation. Phosphorylated IRF3 homo-dimerizes and translocates into the nucleus for transcription of Type I IFN genes. RIG-I or RIG-I like receptors are expressed in all nucleated cells, and they recognizes viral RNA found in the cytoplasm. Upon activation, RIG-I recruits MAVS through the CARD domain interaction, and, analogous to TRIF, MAVS further binds IKKε, TBK1 and IRF3 to promote IRF3-activation and Type I IFN expression.

In contrast to pDCs, the role of TLR7 and TLR8 in cDCs predominantly initiates a DC maturation program that leads to the production of IL-12 rather than Type I IFN (Ito, 2002; Larangé et al., 2009). Type I IFN production by cDC is primarily triggered through the activation of TLR3 that recognizes viral-derived double stranded RNA and TLR4 that recognizes the gram negative bacterial cell wall component lipopolysaccharide. TLR3 is the only TLR that exclusively recruits Trif to mediate signaling, while TLR4 signals through both MyD88 and Trif (Weighardt et al., 2004). Trif is the key adaptor molecule that mediates Type I IFN expression downstream of both receptors, and upon activation, Trif binds the TNF receptor-associated factor 3 (TRAF3) and TRAF6, which recruits RIP1 that induces NFκB activation. Conversely, TRAF3 becomes polyubiquitinated to the lysine at position 63 of the ubiquitin molecule (K63-linkage). K63-linked TRAF3 is essential for the recruitment of TBK1, IKKε, and IRF3, ultimately leading to IRF3 phosphorylation (Oganesyan et al., 2005; Tseng et al., 2010; Häcker et al., 2011). Phoshporylated IRF3 subsequently homo-dimerizes and translocates into the nucleus. Together IRF3 and NFκB form the transcription factors required for the expression of IFN genes (Figure 1). Taken together, pDCs and cDCs have unique roles in response to infection. The expression of different PRRs and differences in the signaling that mediate Type I IFN production in pDCs versus cDCs conspire to contain infection by triggering a systemic antiviral state and efficiently priming T cell activation.

### RIG-I or RIG-I like receptors

In contrast to TLRs which are primarily expressed on innate immune cells, RIG-I are ubiquitously expressed in the cytoplasm of all nucleated cells. Instead of actively sensing viral particles, RIG-I are triggered when cells become infected. RIG-I and the RIG-I like receptors, such as the melanoma differentiation antigen 5 (MDA5) belong to a family of DExD/H box RNA helicases. The N-terminal region of RIG-I is characterized by two caspase recruitment domains (CARD), and the C-terminal region contains RNA helicase activity (Yoneyama and Fujita, 2009). RIG-I recognizes double stranded RNA by the RNA helicase domain, and through a CARD–CARD interaction, RIG-I recruits the CARD-containing adaptor MAVS (also known as VISA, IPS-1, or CARDIF) to mediate downstream events (Yoneyama and Fujita, 2009). Upon activation, MAVS localizes on sub-cellular compartments including the mitochondrial membrane and peroxisomes. Signaling through the peroxisomal-localized MAVS leads to rapid induction of antiviral genes but independent of Type I IFN production (Dixit et al., 2010). In contrast, mitochondrial MAVS produces a slower kinetic that results in Type I IFN expression. Using the mitochondria membrane as a scaffold and powered by the mitochondrial membrane potential (Koshiba et al., 2011), MAVS interacts with TRAF3, TBK1, and IKKε to form a “signalosome” that phosphorylates IRF3 and IRF7 for Type I IFN production (Kawai et al., 2005; Seth et al., 2005). *In vivo* models have shown that RIG-I-deficient mice, despite having intact TLR signaling, succumb to infection by vesicular stomatitis virus, Newcastle disease virus, and Sendai virus (Kato et al., 2005). TLRs and RIG-I signaling complement each other to provide complete coverage across various types of viruses, and both detection systems are geared toward rapid production of Type I IFN that leads to a systemic antiviral state and the control of viral infection.

## Induction of Type I IFN by the TNFR Family

The TNFRSF is a very diverse family of receptors comprising of 29 different family members that provide critical signals regulating numerous physiological functions including inflammation, lymphoid organ development, and adaptive immune responses (Force et al., 1995; Sarin et al., 1995; Lin and Stavnezer, 1996). More recently, it has been shown that TNFR-1 and LTβR activation can trigger Type I IFN expression in macrophages and in cDCs respectively (Yarilina et al., 2008; Summers-deLuca et al., 2011). The production of Type I IFN by TNFR family members in the absence of PRR signaling signifies a novel mechanism of Type I IFN induction that may be critical during immune responses to non-replicating antigen such as tumor antigens and self-antigens.

### TNFR-1 and Type I IFN

**Figure 2:**
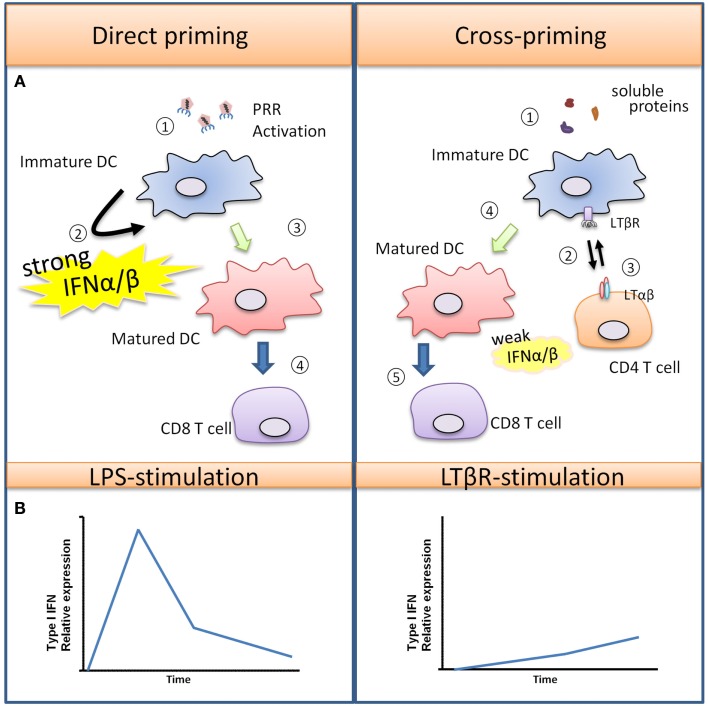
**Type I IFN promotes T cell priming during viral infection versus soluble antigens**. **(A)** Viruses can trigger PRR activation on immature DCs, leading to DC maturation and production of various pro-inflammatory cytokines and a large quantity of Type I IFN. In this scenario, DCs are strongly activated, and they are capable of directly interacting with CD8+ T cells for the generation of virus-specific CTLs. In the case of soluble antigens, DCs are poorly activated due to the absence of PRR-stimulus. Semi-mature DCs must first interact with helper CD4+ helper T cells which rapidly up-regulate “help signals” CD40L and LTαβ upon activation. DC-intrinsic LTβR and CD40 activation promotes DC maturation, with LTβR signaling producing a modest amount of Type I IFN for a sustained period that facilitates CD8+ T cell expansion. **(B)** The differences in kinetics and magnitude of Type I IFN induced by PRR or TNFSFR are illustrated graphically here.

TNF-α was first discovered to be an endotoxin-induced serum factor that exerts cytotoxic effects against sarcoma cells in mice (Carswell et al., 1975), and it has since been recognized as a pleiotropic cytokine that regulates numerous biological functions. Macrophages are major producers of TNF-α in both acute immune responses and chronic inflammatory diseases. TNF-α induces inflammation by affecting many different cell types throughout the body due to the ubiquitous expression of two distinct receptors, TNFR-1 and TNFR-2. Most biological functions are mediated through TNFR-1, while TNFR-2 has been shown to potentiate TNFR-1 induced cell death (Li et al., 2002). TNF-α stimulation in primary human and mouse macrophages induces the phosphorylation of IRF1 and IRF3 leading to the production of Type I IFN and the secretion of chemokines, such as CXCL10 and CXCL11 (Yarilina et al., 2008). Activated T cells express the chemokine receptor CXCR3 that binds to CXCL9, CXCL10, and CXCL11, resulting in their recruitment into sites of inflammation, and numerous studies have recognized the critical role of CXCR3 activating chemokines in various immune responses such as autoimmune mediated skin inflammation and allograft rejection that lack overt PRR activation (Flier et al., 2001; Meyer et al., 2001; Zhao et al., 2002). What is important to note is that the modest and gradual kinetics of the TNFR-1 induced Type I IFN response described above contrast with the more rapid and robust Type I IFN induced by PRR mediated responses such as what occurs during infection – see Figure 2 (Sakaguchi et al., 2003; Honda et al., 2005; Seth et al., 2005).

### LTβR and Type I IFN

LTβR is expressed on stromal cells, DCs, macrophages, and high endothelial venules, while the ligand LTαβ is found on lymphoid tissue inducer cells, B cells and activated T cells. LTβR signaling in stromal cells is required for the development of SLOs (Murphy et al., 1998; Mebius, 2003), and it also plays an important role in the homeostatic maintenance of specific subsets of cDCs in the spleen (Kabashima et al., 2005; Wang et al., 2005; De Trez et al., 2008). More recently, our lab as well as others have examined the role of LTβR in DC: T cell cross-talk (Summers-DeLuca et al., 2007; León et al., 2012). During an immune response, activated CD4+ helper T cells up-regulate CD40L and LTαβ which binds to the corresponding receptors CD40 and LTβR respectively on cDCs. Both CD40 and LTβR signaling provide “help” for cDCs so that they may optimally cross-present antigen for CD8+ T cell activation. Using an immunization model of cell-associated protein antigen, we found that LTβR signaling in cDCs is required for optimal CD8+ T cell clonal expansion while CD40 signaling was required for CD8+ T cell effector functions. Since Benedict and colleagues demonstrated that LTβR signaling could induce Type I IFN in stromal cells, independent of PRR activation (Schneider et al., 2008), we also explored whether direct LTβR stimulation leads to Type I IFN induction in DCs. We found that, not unlike the scenario for TNFR-1, LTβR stimulation induced a modest level of Type I IFN that gradually increased over time. Furthermore, LTβR signaling was required for maximal Type I IFN production in the context of LPS (TLR4) co-stimulation (Summers-deLuca et al., 2011).

Interestingly, the modest level of Type I IFN induced by LTβR signaling in cDCs was important for optimizing CD8+ T cell clonal expansion *in vitro*, since low amounts of exogenous IFN-α can rescue CD8+ T cell expansion in the absence of LTβR signaling. Hence, Type I IFN produced by LTβR or TNFR-1 may facilitate optimal T cell recruitment and/or clonal expansion (Figure 2). Given that some autoimmune diseases are characterized by a “Type I IFN signature” (Lande et al., 2007; Sozzani et al., 2010; Elkon and Stone, 2011), it will be of interest to determine the relevancy of these PRR-independent signals for the induction of Type I IFN in the context of autoimmunity.

## The Many Roles of Type I IFN

Thus far, we have described two very different mechanisms for Type I IFN induction in DCs and monocytes. PRRs detect viral nucleic acids, to produce a robust Type I IFN response for viral clearance, and this is particularly the case for pDCs. Conversely, TNFRSF (TNFR-I, LTβR) induce a modest but prolonged expression of Type I IFN that acts in a co-stimulatory manner to potentiate T cell-mediated immunity (Figure 2B). Therefore, the difference in level, period, and duration of Type I IFN produced by DCs likely dictate the pleiotropic effects of this cytokine resulting in pro-inflammatory or pro-regulatory immune functions.

### Pro-inflammatory function of Type I IFN

Type I IFN is generally regarded as a pro-inflammatory cytokine in numerous immune settings such as autoimmune diseases like psoriasis and systemic lupus erythematosus (Nestle et al., 2005; Lande et al., 2007; Elkon and Stone, 2011), allograft rejection in transplantation (Tovey et al., 1996), and immunity against tumors (Diamond et al., 2011; Fuertes et al., 2011). Type I IFN has robust pro-inflammatory effects that can act in both an autocrine and paracrine manner on immune cells to modulate their functions. During an immune response with minimal PRR activation, DCs cannot reach maximal immunogenic status, and Type I IFN overcomes this hurdle by promoting DC maturation (Le Bon and Tough, 2008; Longhi et al., 2009; Axtell et al., 2013). IFNAR activation in DC triggers NFκB and p38 mitogen-activated protein kinase (MAPK) activation, resulting in the up-regulation of MHC class I and class II as well as co-stimulatory molecules B7-H1 and B7-H2 (Pollara et al., 2006). In addition, blood circulating monocytes, when differentiated into DCs in the presence of Type I IFN, upregulate the chemokine receptor CCR7 thus allowing them to migrate more efficiently into SLOs (Parlato, 2001). In human CD4+ T cells, Type I IFN can complement IL-12 to drive TH-1 differentiation, where IFNAR-mediated STAT2 phosphorylation recruits and activates STAT4, a transcription factor that potentiates IL-12R signaling (Farrar et al., 2000; Gautier et al., 2005). Moreover, Type I IFN signaling through STAT4 has also been implicated in the induction of IFN-γ in natural killer cells and T cells, particularly in the absence of STAT1 (Nguyen et al., 2000). Various studies have reported that Type I IFN acts as a potent third signal that promotes antigen cross-priming (Curtsinger et al., 2005; Le Bon et al., 2006; Le Bon and Tough, 2008). IFNAR activation in CD8+ T cell can induce chromatin remodeling through histone acetelyation, promoting transcription of many genes required for clonal expansion and production of effector molecules (Agarwal et al., 2009). Lastly, Type I IFN prolongs the CD8+ T cell expansion phase in response to cross-presented antigen, and it enhances the responsiveness of antigen specific CD8+ T cells to IL-2 and IL-15 for increased survival (Le Bon et al., 2006). Hence, Type I IFN can independently act on DCs, CD4+ T cells, and CD8+ T cells through very different mechanisms that facilitate inflammatory immune responses.

### Anti-inflammatory role of Type I IFN

Many experimental and clinical settings use Type I IFN as a treatment to quiet inflammatory conditions, suggesting that Type I IFN can exert immunoregulatory functions. In particular, IFN-β has been shown to be an effective therapeutic treatment for collagen-induced arthritis (Van Holten et al., 2004; Adriaansen et al., 2006), relapsing-remitting multiple sclerosis (MS) (Weinstock-Guttman et al., 2008) and autoimmune familial Mediterranean fever (Tweezer-Zaks et al., 2008; Guarda et al., 2011). The anti-inflammatory functions of Type I IFN, particularly in MS, have been characterized by numerous studies, and yet the exact mechanism remains unclear. Blood-derived DCs become activated upon IFNAR activation, however astrocytes and microglia in the central nervous system (CNS) down-regulate MHC-II in response to Type I IFN (Satoh et al., 1995; Hall et al., 1997). T cell recruitment into the CNS may require Type I IFN to induce relevant CXCR3 attracting chemokines, however other studies have also showed that prolonged IFN-β treatment in MS patients down-regulates the expression of cell adhesion molecules such as VCAM-1 and ICAM-1 in brain endothelial cells, resulting in reduced immune cell infiltration into the CNS (Corsini et al., 1997; Defazio et al., 2000). In addition, IFN-β can prevent leukocyte egress from lymph nodes by down-regulating the sphingosine 1-phosphate receptor-1 (S1P1) (Shiow et al., 2006; Gao et al., 2009), and S1P receptor agonists are used to treat MS (Kataoka et al., 2005; Chun and Hartung, 2010; Choi et al., 2011; Galicia-Rosas et al., 2012). IFN-β or IFNAR1 deficient mice have been shown to produce an enhanced number of antigen specific CD8+ T cells when immunized with a DNA-based vaccine, suggesting that Type I IFN is also required to control T cell proliferation (Dikopoulos et al., 2005). Recent studies showed that IFN-induced STAT1 activation negatively regulates the expression and function of the oncogene c-myc in CD8+ T cells which is important for homeostatic proliferation (Gil et al., 2006, 2012). Furthermore, other studies have also shown that IFNAR or IFN-β deficient mice exhibit lower numbers of IL-10 producing T cells, which may also explain the increased CD8 T cell expansion in the absence of IFNAR signaling (Dikopoulos et al., 2005; Bochtler et al., 2008). It is important to point out, however, that scenarios where IFNAR signaling is completely absent may lead to different effects on shaping T cell responses than situations where the levels/kinetics of Type I IFN production have been altered.

## Conclusions

Type I IFN is a pleiotropic cytokine that affects many different cell types with a wide range of effects. The modulation of the innate and adaptive immune responses relies on a finely tuned rheostat of Type I IFN production. The amount of IFN produced through a particular time frame during the course of an immune reaction will likely dictate very different outcomes. Therefore, understanding the mechanisms underlying Type I IFN induction by DCs is crucial for providing future groundwork in developing therapeutics that quiet autoimmunity or promote pathogen and tumour clearance.

## Conflict of Interest Statement

The authors declare that the research was conducted in the absence of any commercial or financial relationships that could be construed as a potential conflict of interest.

## References

[B1] Adriaansen J. Kuhlman R. R. Van Holten J. Kaynor C. Vervoordeldonk M. J. B. M. Tak P. P. (2006). Intraarticular interferon-beta gene therapy ameliorates adjuvant arthritis in rats. Hum. Gene Ther. 17, 985–996 10.1089/hum.2006.17.985 16984225

[B2] Agarwal P. Raghavan A. Nandiwada S. L. Curtsinger J. M. Bohjanen P. R. Mueller D. L. (2009). Gene regulation and chromatin remodeling by IL-12 and type I IFN in programming for CD8 T cell effector function and memory. J. Immunol. 183, 1695–1704 10.4049/jimmunol.0900592 19592655 PMC2893405

[B3] Akira S. (2011). Innate immunity and adjuvants. Philos. Trans. R. Soc. Lond. B Biol. Sci. 366, 2748–2755 10.1098/rstb.2011.0106 21893536 PMC3146784

[B4] Asselin-Paturel C. Trinchieri G. (2005). Production of type I interferons: plasmacytoid dendritic cells and beyond. J. Exp. Med. 202, 461–465 10.1084/jem.20051395 16103406 PMC2212850

[B5] Axtell R. C. Raman C. Steinman L. (2013). Type I interferons: beneficial in Th1 and detrimental in Th17 autoimmunity. Clin. Rev. Allergy Immunol. 44, 114–120 10.1007/s12016-011-8296-5 22231516 PMC5478162

[B6] Barton G. M. Kagan J. C. Medzhitov R. (2006). Intracellular localization of toll-like receptor 9 prevents recognition of self DNA but facilitates access to viral DNA. Nat. Immunol. 7, 49–56 10.1038/ni1280 16341217

[B7] Bochtler P. Kröger A. Schirmbeck R. Reimann J. (2008). Type I IFN-induced, NKT cell-mediated negative control of CD8 T cell priming by dendritic cells. J. Immunol. 181, 1633–1643 18641299 10.4049/jimmunol.181.3.1633

[B8] Carbone F. R. Bevan M. J. (1990). Class I-restricted processing and presentation of exogenous cell-associated antigen in vivo. J. Exp. Med. 171, 377–387 10.1084/jem.171.2.377 2137512 PMC2187713

[B9] Carswell E. A. Old L. J. Kassel R. L. Green S. Fiore N. Williamson B. (1975). An endotoxin-induced serum factor that causes necrosis of tumors. Proc. Natl. Acad. Sci. U.S.A. 72, 3666–3670 10.1073/pnas.72.9.3666 1103152 PMC433057

[B10] Casanova J.-L. Abel L. Quintana-Murci L. (2011). Human TLRs and IL-1Rs in host defense: natural insights from evolutionary, epidemiological, and clinical genetics. Annu. Rev. Immunol. 29, 447–491 10.1146/annurev-immunol-030409-101335 21219179

[B11] Choi J. W. Gardell S. E. Herr D. R. Rivera R. Lee C.-W. Noguchi K. (2011). FTY720 (fingolimod) efficacy in an animal model of multiple sclerosis requires astrocyte sphingosine 1-phosphate receptor 1 (S1P1) modulation. Proc. Natl. Acad. Sci. U.S.A. 108, 751–756 10.1073/pnas.1007566108 21177428 PMC3021041

[B12] Chun J. Hartung H.-P. (2010). Mechanism of action of oral fingolimod (FTY720) in multiple sclerosis. Clin. Neuropharmacol. 33, 91–101 10.1097/WNF.0b013e3181cbf825 20061941 PMC2859693

[B13] Coccia E. M. Severa M. Giacomini E. Monneron D. Remoli M. E. Julkunen I. (2004). Viral infection and toll-like receptor agonists induce a differential expression of type I and lambda interferons in human plasmacytoid and monocyte-derived dendritic cells. Eur. J. Immunol. 34, 796–805 10.1002/eji.200324610 14991609

[B14] Colonna M. Trinchieri G. Liu Y.-J. (2004). Plasmacytoid dendritic cells in immunity. Nat. Immunol. 5, 1219–1226 10.1038/ni1141 15549123

[B15] Corsini E. Gelati M. Dufour A. Massa G. Nespolo A. Ciusani E. (1997). Effects of beta-IFN-1b treatment in MS patients on adhesion between PBMNCs, HUVECs and MS-HBECs: an in vivo and in vitro study. J. Neuroimmunol. 79, 76–83 10.1016/S0165-5728(97)00114-8 9357450

[B16] Curtsinger J. J. M. Valenzuela J. J. O. Agarwal P. Lins D. Mescher M. F. (2005). Type I IFNs provide a third signal to CD8 T cells to stimulate clonal expansion and differentiation. J. Immunol. 174, 4465–4469 15814665 10.4049/jimmunol.174.8.4465

[B17] Dalgaard J. Beckstrøm K. J. Jahnsen F. L. Brinchmann J. E. (2005). Differential capability for phagocytosis of apoptotic and necrotic leukemia cells by human peripheral blood dendritic cell subsets. J. Leukoc. Biol. 77, 689–698 10.1189/jlb.1204711 15728242

[B18] De Trez C. Schneider K. Potter K. Droin N. Fulton J. Norris P. S. (2008). The inhibitory HVEM-BTLA pathway counter regulates lymphotoxin receptor signaling to achieve homeostasis of dendritic cells. J. Immunol. 180, 238–248 18097025 10.4049/jimmunol.180.1.238 PMC2711003

[B19] Defazio G. Livrea P. Giorelli M. Martino D. Roselli F. Ricchiuti F. (2000). Interferon beta-1a downregulates TNFalpha-induced intercellular adhesion molecule 1 expression on brain microvascular endothelial cells through a tyrosine kinase-dependent pathway. Brain Res. 881, 227–230 10.1016/S0006-8993(00)02814-6 11036165

[B20] Summers-deLuca L. Ng D. Gao Y. Wortzman M. E. Watts T. H. Gommerman J. L. (2011). LTβR signaling in dendritic cells induces a type I IFN response that is required for optimal clonal expansion of CD8+ T cells. Proc. Natl. Acad. Sci. U.S.A. 108, 2046–2051 10.1073/pnas.1014188108 21245292 PMC3033245

[B21] Den Haan J. M. Lehar S. M. Bevan M. J. (2000). CD8(+) but not CD8(-) dendritic cells cross-prime cytotoxic T cells in vivo. J. Exp. Med. 192, 1685–1696 10.1084/jem.192.12.1685 11120766 PMC2213493

[B22] Diamond M. S. Kinder M. Matsushita H. Mashayekhi M. Dunn G. P. Archambault J. M. (2011). Type I interferon is selectively required by dendritic cells for immune rejection of tumors. J. Exp. Med. 208, 1989–2003 10.1084/jem.20101158 21930769 PMC3182061

[B23] Dikopoulos N. Bertoletti A. Kröger A. Hauser H. Schirmbeck R. Reimann J. (2005). Type I IFN negatively regulates CD8+ T cell responses through IL-10-producing CD4+ T regulatory 1 cells. J. Immunol. 174, 99–109 15611232 10.4049/jimmunol.174.1.99

[B24] Dixit E. Boulant S. Zhang Y. Lee A. S. Y. Odendall C. Shum B. (2010). Peroxisomes are signaling platforms for antiviral innate immunity. Cell 141, 668–681 10.1016/j.cell.2010.04.018 20451243 PMC3670185

[B25] Elkon K. B. Stone V. V. (2011). Type I interferon and systemic lupus erythematosus. J. Interferon Cytokine Res. 31, 803–812 10.1089/jir.2011.0045 21859344 PMC3216059

[B26] Farrar J. D. Smith J. D. Murphy T. L. Murphy K. M. (2000). Recruitment of Stat4 to the human interferon-alpha/beta receptor requires activated Stat2. J. Biol. Chem. 275, 2693–2697 10.1074/jbc.M003399200 10644731

[B27] Flier J. Boorsma D. M. Van Beek P. J. Nieboer C. Stoof T. J. Willemze R. (2001). Differential expression of CXCR3 targeting chemokines CXCL10, CXCL9, and CXCL11 in different types of skin inflammation. J. Pathol. 194, 398–405 10.1002/1096-9896(200108)194:4<397::AID-PATH899>3.0.CO;2-S 11523046

[B28] Force W. R. Walter B. N. Hession C. Tizard R. Kozak C. A. Browning J. L. (1995). Mouse lymphotoxin-beta receptor. Molecular genetics, ligand binding, and expression. J. Immunol. 155, 5280–5288 7594541

[B29] Fuertes M. B. Kacha A. K. Kline J. Woo S.-R. Kranz D. M. Murphy K. M. (2011). Host type I IFN signals are required for antitumor CD8+ T cell responses through CD8+ dendritic cells. J. Exp. Med. 208, 2005–2016 10.1084/jem.20101159 21930765 PMC3182064

[B30] Galicia-Rosas G. Pikor N. Schwartz J. A. Rojas O. Jian A. Summers-Deluca L. (2012). A sphingosine-1-phosphate receptor 1-directed agonist reduces central nervous system inflammation in a plasmacytoid dendritic cell-dependent manner. J. Immunol. 189, 3700–3706 10.4049/jimmunol.1102261 22933630

[B31] Gao Y. Majchrzak-Kita B. Fish E. N. Gommerman J. L. (2009). Dynamic accumulation of plasmacytoid dendritic cells in lymph nodes is regulated by interferon-beta. Blood 114, 2623–2631 10.1182/blood-2008-10-183301 19652204

[B32] Gautier G. Humbert M. Deauvieau F. Scuiller M. Hiscott J. Bates E. E. M. (2005). A type I interferon autocrine-paracrine loop is involved in toll-like receptor-induced interleukin-12p70 secretion by dendritic cells. J. Exp. Med. 201, 1435–1446 10.1084/jem.20041964 15851485 PMC2213193

[B33] Gil M. Salomon R. Louten J. Biron C. (2006). Modulation of STAT1 protein levels: a mechanism shaping CD8 T-cell responses in vivo. Blood 107, 987–993 10.1182/blood-2005-07-2834 16210337 PMC1895900

[B34] Gil M. P. Ploquin M. J. Y. Watford W. T. Lee S.-H. Kim K. Wang X. (2012). Regulating type 1 IFN effects in CD8 T cells during viral infections: changing STAT4 and STAT1 expression for function. Blood 120, 3718–3728 10.1182/blood-2012-05-428672 22968462 PMC3488885

[B35] Guarda G. Braun M. Staehli F. Tardivel A. Mattmann C. Förster I. (2011). Type I interferon inhibits interleukin-1 production and inflammasome activation. Immunity 34, 213–223 10.1016/j.immuni.2011.02.006 21349431

[B36] Häcker H. Tseng P. Karin M. (2011). Expanding TRAF function: TRAF3 as a tri-faced immune regulator. Nat. Rev. Immunol. 11, 457–468 10.1038/nri2998 21660053

[B37] Hall G. L. Wing M. G. Compston D. A. Scolding N. J. (1997). β-interferon regulates the immunomodulatory activity of neonatal rodent microglia. J. Neuroimmunol. 72, 11–19 10.1016/S0165-5728(96)00128-2 9003241

[B38] Hashimoto D. Miller J. Merad M. (2011). Dendritic cell and macrophage heterogeneity in vivo. Immunity 35, 323–335 10.1016/j.immuni.2011.09.007 21943488 PMC4520532

[B39] Honda K. Yanai H. Negishi H. Asagiri M. (2005). IRF-7 is the master regulator of type-I interferon-dependent immune responses. Nature 434, 772–777 10.1038/nature03547 15800576

[B40] Hornung V. Rothenfusser S. Britsch S. Krug A. Jahrsdörfer B. Giese T. (2002). Quantitative expression of toll-like receptor 1-10 mRNA in cellular subsets of human peripheral blood mononuclear cells and sensitivity to CpG oligodeoxynucleotides. J. Immunol. 168, 4531–4537 11970999 10.4049/jimmunol.168.9.4531

[B41] Ito T. (2002). Interferon-alpha and interleukin-12 are induced differentially by toll-like receptor 7 ligands in human blood dendritic cell subsets. J. Exp. Med. 195, 1507–1512 10.1084/jem.20020207 12045249 PMC2193542

[B42] Kabashima K. Banks T. A. Ansel K. M. Lu T. T. Ware C. F. Cyster J. G. (2005). Intrinsic lymphotoxin-beta receptor requirement for homeostasis of lymphoid tissue dendritic cells. Immunity 22, 439–450 10.1016/j.immuni.2005.02.007 15845449

[B43] Kataoka H. Sugahara K. Shimano K. Teshima K. Koyama M. Fukunari A. (2005). FTY720, sphingosine 1-phosphate receptor modulator, ameliorates experimental autoimmune encephalomyelitis by inhibition of T cell infiltration. Cell. Mol. Immunol. 2, 439–448 16426494

[B44] Kato H. Sato S. Yoneyama M. Yamamoto M. Uematsu S. Matsui K. (2005). Cell type-specific involvement of RIG-I in antiviral response. Immunity 23, 19–28 10.1016/j.immuni.2005.04.010 16039576

[B45] Kawai T. Takahashi K. Sato S. Coban C. Kumar H. Kato H. (2005). IPS-1, an adaptor triggering RIG-I- and Mda5-mediated type I interferon induction. Nat. Immunol. 6, 981–988 10.1038/ni1243 16127453

[B46] Koshiba T. Yasukawa K. Yanagi Y. Kawabata S. (2011). Mitochondrial membrane potential is required for MAVS-mediated antiviral signaling. Sci. Signal. 4, ra7 10.1126/scisignal.2001147 21285412

[B47] Lande R. Gregorio J. Facchinetti V. Chatterjee B. Wang Y.-H. Homey B. (2007). Plasmacytoid dendritic cells sense self-DNA coupled with antimicrobial peptide. Nature 449, 564–569 10.1038/nature06116 17873860

[B48] Larangé A. Antonios D. Pallardy M. Kerdine-Römer S. (2009). TLR7 and TLR8 agonists trigger different signaling pathways for human dendritic cell maturation. J. Leukoc. Biol. 85, 673–683 10.1189/jlb.0808504 19164127

[B49] Le Bon A. Durand V. Kamphuis E. Thompson C. Bulfone-Paus S. Rossmann C. (2006). Direct stimulation of T cells by type I IFN enhances the CD8+ T cell response during cross-priming. J. Immunol. 176, 4682–4689 16585561 10.4049/jimmunol.176.8.4682

[B50] Le Bon A. Tough D. F. (2008). Type I interferon as a stimulus for cross-priming. Cytokine Growth Factor Rev. 19, 33–40 10.1016/j.cytogfr.2007.10.007 18068417

[B51] León B. Ballesteros-Tato A. Browning J. L. Dunn R. Randall T. D. Lund F. E. (2012). Regulation of T(H)2 development by CXCR5+ dendritic cells and lymphotoxin-expressing B cells. Nat. Immunol. 13, 681–690 10.1038/ni.2309 22634865 PMC3548431

[B52] Li X. Yang Y. Ashwell J. D. (2002). TNF-RII and c-IAP1 mediate ubiquitination and degradation of TRAF2. Nature 416, 345–347 10.1038/416345a 11907583

[B53] Lin S. C. Stavnezer J. (1996). Activation of NF-kappaB/Rel by CD40 engagement induces the mouse germ line immunoglobulin Cgamma1 promoter. Mol. Cell. Biol. 16, 4591–4603 8756615 10.1128/mcb.16.9.4591 PMC231458

[B54] Longhi M. P. Trumpfheller C. Idoyaga J. Caskey M. Matos I. Kluger C. (2009). Dendritic cells require a systemic type I interferon response to mature and induce CD4+ Th1 immunity with poly IC as adjuvant. J. Exp. Med. 206, 1589–1602 10.1084/jem.20090247 19564349 PMC2715098

[B55] Mebius R. E. (2003). Organogenesis of lymphoid tissues. Nat. Rev. Immunol. 3, 292–303 10.1038/nri1054 12669020

[B56] Meyer M. Hensbergen P. J. Van der Raaij-Helmer E. M. Brandacher G. Margreiter R. Heufler C. (2001). Cross reactivity of three T cell attracting murine chemokines stimulating the CXC chemokine receptor CXCR3 and their induction in cultured cells and during allograft rejection. Eur. J. Immunol. 31, 2521–2527 10.1002/1521-4141(200108)31:8<2521::AID-IMMU2521>3.0.CO;2-Q 11500837

[B57] Meylan E. Tschopp J. (2006). Toll-like receptors and RNA helicases: two parallel ways to trigger antiviral responses. Mol. Cell 22, 561–569 10.1016/j.molcel.2006.05.012 16762830

[B58] Murphy M. Walter B. N. Pike-Nobile L. Fanger N. A. Guyre P. M. Browning J. L. (1998). Expression of the lymphotoxin beta receptor on follicular stromal cells in human lymphoid tissues. Cell Death Differ. 5, 497–505 10.1038/sj.cdd.4400374 10200501

[B59] Nestle F. O. Conrad C. Tun-Kyi A. Homey B. Gombert M. Boyman O. (2005). Plasmacytoid predendritic cells initiate psoriasis through interferon-alpha production. J. Exp. Med. 202, 135–143 10.1084/jem.20050500 15998792 PMC2212894

[B60] Nguyen K. B. Cousens L. P. Doughty L. A. Pien G. C. Durbin J. E. Biron C. A. (2000). Interferon alpha/beta-mediated inhibition and promotion of interferon gamma: STAT1 resolves a paradox. Nat. Immunol. 1, 70–76 10.1038/76940 10881178

[B61] Oganesyan G. Saha S. Guo B. He J. (2005). Critical role of TRAF3 in the toll-like receptor-dependent and-independent antiviral response. Nature 439, 208–211 10.1038/nature04374 16306936

[B62] Parlato S. (2001). Expression of CCR-7, MIP-3beta, and Th-1 chemokines in type I IFN-induced monocyte-derived dendritic cells: importance for the rapid acquisition of potent migratory and functional activities. Blood 98, 3022–3029 10.1182/blood.V98.10.3022 11698286

[B63] Platanias L. C. (2005). Mechanisms of type-I- and type-II-interferon-mediated signalling. Nat. Rev. Immunol. 5, 375–386 10.1038/nri1604 15864272

[B64] Pollara G. Handley M. E. Kwan A. Chain B. M. Katz D. R. (2006). Autocrine type I interferon amplifies dendritic cell responses to lipopolysaccharide via the nuclear factor-kappaB/p38 pathways. Scand. J. Immunol. 63, 151–154 10.1111/j.1365-3083.2006.01727.x 16499567

[B65] Pooley J. Heath W. Shortman K. (2001). Cutting edge: intravenous soluble antigen is presented to CD4 T cells by CD8-dendritic cells, but cross-presented to CD8 T cells by CD8+ dendritic cells. J. Immunol. 166, 5327–5330 11313367 10.4049/jimmunol.166.9.5327

[B66] Reynolds J. M. Pappu B. P. Peng J. Martinez G. J. Zhang Y. Chung Y. (2010). Toll-like receptor 2 signaling in CD4(+) T lymphocytes promotes T helper 17 responses and regulates the pathogenesis of autoimmune disease. Immunity 32, 692–702 10.1016/j.immuni.2010.04.010 20434372 PMC2878917

[B67] Sakaguchi S. Negishi H. Asagiri M. Nakajima C. Mizutani T. Takaoka A. (2003). Essential role of IRF-3 in lipopolysaccharide-induced interferon-β gene expression and endotoxin shock. Biochem. Biophys. Res. Commun. 306, 860–866 10.1016/S0006-291X(03)01049-0 12821121

[B68] Sapoznikov A. Fischer J. A. Zaft T. Krauthgamer R. Dzionek A. Jung S. (2007). Organ-dependent in vivo priming of naive CD4+, but not CD8+, T cells by plasmacytoid dendritic cells. J. Exp. Med. 204, 1923–1933 10.1084/jem.20062373 17646404 PMC2118686

[B69] Sarin A. Conan-Cibotti M. Henkart P. A. (1995). Cytotoxic effect of TNF and lymphotoxin on T lymphoblasts. J. Immunol. 155, 3716–3718 7561073

[B70] Satoh J. Paty D. W. Kim S. U. (1995). Differential effects of beta and gamma interferons on expression of major histocompatibility complex antigens and intercellular adhesion molecule-1 in cultured fetal human astrocytes. Neurology 45, 367–373 10.1212/WNL.45.2.367 7854540

[B71] Schneider K. Loewendorf A. De Trez C. Fulton J. Rhode A. Shumway H. (2008). Lymphotoxin-mediated crosstalk between B cells and splenic stroma promotes the initial type I interferon response to cytomegalovirus. Cell Host Microbe 3, 67–76 10.1016/j.chom.2007.12.008 18312841 PMC2703178

[B72] Sen G. C. (2001). Viruses and interferons. Annu. Rev. Microbiol. 55, 255–281 10.1146/annurev.micro.55.1.255 11544356

[B73] Seth R. B. Sun L. Ea C.-K. Chen Z. J. (2005). Identification and characterization of MAVS, a mitochondrial antiviral signaling protein that activates NF-kappaB and IRF 3. Cell 122, 669–682 10.1016/j.cell.2005.08.012 16125763

[B74] Shiow L. R. Rosen D. B. Brdicková N. Xu Y. An J. Lanier L. L. (2006). CD69 acts downstream of interferon-alpha/beta to inhibit S1P1 and lymphocyte egress from lymphoid organs. Nature 440, 540–544 10.1038/nature04606 16525420

[B75] Siegal F. P. (1999). The nature of the principal type 1 interferon-producing cells in human blood. Science 284, 1835–1837 10.1126/science.284.5421.1835 10364556

[B76] Sozzani S. Bosisio D. Scarsi M. Tincani A. (2010). Type I interferons in systemic autoimmunity. Autoimmunity 43, 196–203 10.3109/08916930903510872 20298124

[B77] Stetson D. B. Medzhitov R. (2006). Type I interferons in host defense. Immunity 25, 373–381 10.1016/j.immuni.2006.08.007 16979569

[B78] Summers-DeLuca L. E. McCarthy D. D. Cosovic B. Ward L. A. Lo C. C. Scheu S. (2007). Expression of lymphotoxin-alphabeta on antigen-specific T cells is required for DC function. J. Exp. Med. 204, 1071–1081 10.1084/jem.20061968 17452522 PMC2118582

[B79] Takeda K. Akira S. (2005). Toll-like receptors in innate immunity. Int. Immunol. 17, 1–14 10.1093/intimm/dxh186 15585605

[B80] Tovey M. G. Benizri E. Gugenheim J. Bernard G. Eid P. Blanchard B. (1996). Role of the type I interferons in allograft rejection. J. Leukoc. Biol. 59, 512–517 8613698 10.1002/jlb.59.4.512

[B81] Tseng P.-H. Matsuzawa A. Zhang W. Mino T. Vignali D. A. Karin M. (2010). Different modes of ubiquitination of the adaptor TRAF3 selectively activate the expression of type I interferons and proinflammatory cytokines. Nat. Immunol. 11, 70–75 10.1038/ni.1819 19898473 PMC2872790

[B82] Tweezer-Zaks N. Rabinovich E. Lidar M. Livneh A. (2008). Interferon-alpha as a treatment modality for colchicine-resistant familial Mediterranean fever. J. Rheumatol. 35, 1362–1365 18528960

[B83] Van Holten J. Reedquist K. Sattonet-Roche P. Smeets T. J. M. Plater-Zyberk C. Vervoordeldonk M. J. (2004). Treatment with recombinant interferon-beta reduces inflammation and slows cartilage destruction in the collagen-induced arthritis model of rheumatoid arthritis. Arthritis Res. Ther. 6, R239–R249 10.1186/ar1165 15142270 PMC416442

[B84] Villadangos J. A. Schnorrer P. (2007). Intrinsic and cooperative antigen-presenting functions of dendritic-cell subsets in vivo. Nat. Rev. Immunol. 7, 543–555 10.1038/nri2103 17589544

[B85] Wang Y.-G. Kim K. D. Wang J. Yu P. Fu Y.-X. (2005). Stimulating lymphotoxin beta receptor on the dendritic cells is critical for their homeostasis and expansion. J. Immunol. 175, 6997–7002 16272360 10.4049/jimmunol.175.10.6997

[B86] Weighardt H. Jusek G. Mages J. Lang R. Hoebe K. Beutler B. (2004). Identification of a TLR4- and TRIF-dependent activation program of dendritic cells. Eur. J. Immunol. 34, 558–564 10.1002/eji.200324714 14768061

[B87] Weinstock-Guttman B. Ramanathan M. Zivadinov R. (2008). Interferon-beta treatment for relapsing multiple sclerosis. Expert Opin. Biol. Ther. 8, 1435–1447 10.1517/14712598.8.9.1435 18694361

[B88] Yarilina A. Park-Min K.-H. Antoniv T. Hu X. Ivashkiv L. B. (2008). TNF activates an IRF1-dependent autocrine loop leading to sustained expression of chemokines and STAT1-dependent type I interferon-response genes. Nat. Immunol. 9, 378–387 10.1038/ni1576 18345002

[B89] Yoneyama M. Fujita T. (2009). RNA recognition and signal transduction by RIG-I-like receptors. Immunol. Rev. 227, 54–65 10.1111/j.1600-065X.2008.00727.x 19120475

[B90] Young L. J. Wilson N. S. Schnorrer P. Proietto A. Ten Broeke T. Matsuki Y. (2008). Differential MHC class II synthesis and ubiquitination confers distinct antigen-presenting properties on conventional and plasmacytoid dendritic cells. Nat. Immunol. 9, 1244–1252 10.1038/ni.1665 18849989

[B91] Zhao D. X. Hu Y. Miller G. G. Luster A. D. Mitchell R. N. Libby P. (2002). Differential expression of the IFN-gamma-inducible CXCR3-binding chemokines, IFN-inducible protein 10, monokine induced by IFN, and IFN-inducible T cell alpha chemoattractant in human cardiac allografts: association with cardiac allograft vasculopathy and acute rejection. J. Immunol. 169, 1556–1560 12133984 10.4049/jimmunol.169.3.1556

